# Supplementary material for: GiantHunter: accurate detection of giant virus in metagenomic data using reinforcement-learning and Monte Carlo tree search
Source: Bioinformatics. 2025 Jul 15;41(Suppl 1):i30–9. doi: 10.1093/bioinformatics/btaf239 (PMC12261416; doi:10.1093/bioinformatics/btaf239)
Supplement: btaf239_Supplementary_Data [file btaf239_supplementary_data.pdf]

GiantHunter: Supplemental Document

1. DETAILS OF THE TRANSFORMER MODEL

The Transformer model is widely used in Natural Language Processing (NLP) and plays an increasing role in biological sequence analysis due to its powerful capability of capturing contextual information in the corpus. Similarly, we utilize this model to process the sequences of PC IDs, as some genes in NCLDV genomes show significant interaction with each other, which can be the signature of this phylum. Specifically, we set the maximum length of the sentences as 1000 because 96% of complete NCLDV genomes from the RefSeq contain less than 1000 proteins. The output of the whole model is the probability of whether the focal contigs are from NCLDV.

The model has two main components: the embedding layer and the self-attention layer. The embedding layer aims to convert discrete IDs to continuous initial embedding vectors for the sequencing process. Word embedding and positional embedding are included in the embedding layer. The word embedding layer is an $n \times m$ look-up table, where n refers to the size of PCs vocabulary and m represents the embedding dimension. As self-attention process does not consider the order of tokens, positional embedding is adopted here to encode the sequential information of PCs tokens. It takes the index of each token as input and outputs an embedding vector with the same dimension of the word embedding. The detailed process is described in equations:

$$PE_{(pos,2i)} = sin(pos/10000^{2i/d_{model}}) \tag{S1}$$

$$PE_{(pos,2i+1)} = cos(pos/10000^{2i/d_{model}}) \tag{S2}$$

where pos is the position and i is the dimension. As for any fixed offset k,PE_{pos+k} can be represented as a linear function of PE_{pos}, and it is hypothesized that the formulas could allow the model to learn to capture the relative positions [1]. Finally, the word embedding and the positional embedding are simply summed up for each token to get the initial embedding vector, whose dimension is 768 here.

Self-attention is the core mechanism of the Transformer model, relating different positions of a single sequence to compute the representation of each token in the sequence [1]. During the process, the matrix composed of embeddings of each token is firstly put into three different FC layers and then three matrices called Query (Q), Key (K) and Value (V) are obtained. After that the model will adopt self-attention mechanism to find co-occurence of some specific PCs. In practice, we compute the matrix of output as:

$$\text{Attention}(Q,K,V) = \text{softmax}(\frac{QK^T}{\sqrt{d_k}})V \tag{S3}$$

where d_k is the dimension of embedding vectors. In detail, QK^T is to exchange the information of each token and compute the attention matrix, divided by $\sqrt{d_k}$ to prevent gradients drop caused by large input into softmax function. After being normalized by the softmax, the attention matrix is obtained where large elements indicate strong semantic connections of tokens. The value matrix V is then multiplied by the attention matrix to get the final embedding of each token. To capture the various combinations of connections, multi-head attention, a modified version of vanilla attention mechanism, is utilized here. Specifically, we linearly project the queries, keys and values h times with different learnable weights and then perform the attention function on each head in parallel, with residual blocks following to capture more complicated information. In the end, we feed the output of Transformer blocks into classification head composed of two FC layers with sigmoid activation function, getting the final probability the focal contig being NCLDV contig.

2. VISUALIZATION OF TOP PC SELECTED BY THE MCTS

To validate that our proposed MCTS-based negative sampling method effectively selects a subset with a distribution different from the entire dataset, we analyzed the distribution of protein clusters (PCs). Specifically, we counted the number of PCs and sorted them to examine their distributions. The PC distribution for the top 20% of most-visited sequences (identified using MCTS) is shown in Figure S1.

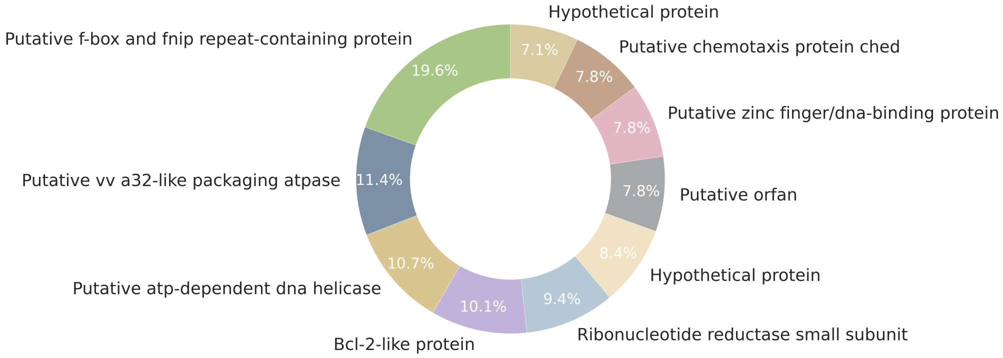

Fig. S1. Top 10 PCs distributions of contigs, where the percentage inside indicates the percentage of counts in the whole top 10 PCs.

The figure highlights several proteins that may provide deeper insights into NCLDVs. For example, A32 is a single-copy marker gene of NCLDVs but ranks second among all bacteriophage-carried PC, suggesting that bacteriophages may also carry this gene. The results showcase the limitations of relying solely on marker genes for identification and may provide insights into the phylogenetic relationships between NCLDVs and bacteriophages. Furthermore, MCTS effectively prioritized challenging examples containing the NCLDV marker gene, demonstrating its utility in selecting difficult cases for training.

3. PROGRAM PARAMETERS

Program	Parameters
Prodigal	default
DIAMOND BLASTP	–evalue 1e-5
mcxload	–stream-mirror –stream-neg-log10 -stream-tf ’ceil(300)’
mcl	-I 2.0

Table S1. The program and parameters used for constructing protein clusters

4. DETAILED PERFORMANCE ON DATA OF DIFFERENT LENGTHS

Length	Precision	Recall	F1-score	Method
5000	0.997628	0.928282	0.961707	GiantHunter
10000	0.998439	0.977082	0.987645	GiantHunter
15000	1.000000	0.987254	0.993586	GiantHunter
20000	1.000000	0.989114	0.994527	GiantHunter
5000	0.989340	0.716808	0.831307	ViralRecall
10000	0.981073	0.950344	0.965464	ViralRecall
15000	0.976852	0.977984	0.977417	ViralRecall
20000	0.972308	0.982893	0.977572	ViralRecall
5000	1.000000	0.498345	0.665194	ViralRecall(Custom)
10000	1.000000	0.741024	0.851251	ViralRecall(Custom)
15000	1.000000	0.822711	0.902734	ViralRecall(Custom)
20000	1.000000	0.846034	0.916596	ViralRecall(Custom)
5000	0.406351	0.785951	0.535723	VirSorter2
10000	0.382800	0.904507	0.537937	VirSorter2
15000	0.363434	0.946698	0.525233	VirSorter2
20000	0.337343	0.959565	0.499191	VirSorter2
5000	0.916258	0.756528	0.828767	VirSorter2(Custom)
10000	0.928403	0.901451	0.914729	VirSorter2(Custom)
15000	0.935632	0.943221	0.939411	VirSorter2(Custom)
20000	0.923308	0.954899	0.938838	VirSorter2(Custom)

Table S2. The performance on different lengths of dataset split by date

Length	Precision	Recall	F1-score	Method
5000	0.996150	0.781722	0.876005	GiantHunter
10000	0.998276	0.881279	0.936136	GiantHunter
15000	1.000000	0.904651	0.949939	GiantHunter
20000	1.000000	0.904321	0.949757	GiantHunter
5000	0.997372	0.573263	0.728058	ViralRecall
10000	0.984645	0.780822	0.870968	ViralRecall
15000	0.973545	0.855814	0.910891	ViralRecall
20000	0.970000	0.898148	0.932692	ViralRecall
5000	1.000000	0.259063	0.411518	ViralRecall(Custom)
10000	1.000000	0.441400	0.612460	ViralRecall(Custom)
15000	1.000000	0.553488	0.712575	ViralRecall(Custom)
20000	1.000000	0.586420	0.739300	ViralRecall(Custom)
5000	0.402534	0.743958	0.522408	VirSorter2
10000	0.381142	0.873668	0.530744	VirSorter2
15000	0.350045	0.909302	0.505495	VirSorter2
20000	0.329741	0.944444	0.488818	VirSorter2
5000	0.913251	0.723565	0.807417	VirSorter2(Custom)
10000	0.927132	0.910198	0.918587	VirSorter2(Custom)
15000	0.906250	0.944186	0.924829	VirSorter2(Custom)
20000	0.896552	0.962963	0.928571	VirSorter2(Custom)

Table S3. The performance on different lengths of dataset split by similarity

5. EXPANDED TRAINING SET

We expanded the negative training set to include all non-NCLDV, non-bacteriophage viruses. Specifically, we downloaded 18,671 complete viral genomes from the NCBI RefSeq database. After excluding NCLDVs and bacteriophages, 13,285 genomes—primarily representing eukaryotic viruses—were retained. Employing the same data augmentation strategy as in prior work, we fragmented these genomes into contigs of 5,000, 10,000, 15,000, and 20,000 base pairs (bp) with 400-bp overlaps. These augmented contigs were added to the training set, while the original validation and test sets remained unmodified to ensure comparability. Due to the Monte Carlo tree search (MCTS) algorithm's sensitivity to random seed initialization, we trained five model instances with distinct seeds. Table S4 summarizes the performance achieved by the expanded training set relative to the original baseline.

	**Recall**	**Precision**	**F1-score**
Original	0.9677 ± 0.0076	0.9937 ± 0.0021	0.9805 ± 0.0034
Expanded	0.9256 ± 0.0034	0.9973 ± 0.0007	0.9601 ± 0.0020

Table S4. Performance metrics for models trained on the original and expanded training sets, evaluated on the NCLDV-phage test set. Results are reported as mean $\pm$ standard deviation.

Contrary to expectations, expanding the training set resulted in diminished model performance, particularly in recall. This decline suggests that the updated model misclassifies certain NCLDV sequences as other viral types. We hypothesize that the newly introduced negative samples disrupted the Monte Carlo tree search (MCTS) process by diluting the pool of challenging negatives. The agent's capacity to prioritize these biologically ambiguous samples during training may have been compromised, leading to reduced specificity. This observation underscores the value of integrating domain-specific knowledge into negative sampling strategies to mitigate such unintended effects.

6. ESM PROTEIN EMBEDDING FEATURES

We evaluated an alternative protein feature extraction strategy by replacing protein cluster (PC) IDs with embeddings derived from the ESM-2 language model. The pipeline begins with translating contigs into protein sequences using Prodigal for gene prediction. These sequences are then processed through the pretrained ESM-2-150M model[2], where token-level embeddings are generated and averaged to produce a single vector representation per protein. Each contig is subsequently represented as an ordered collection of these protein embeddings. To classify contigs, we trained a lightweight single-layer Transformer model on the original NCLDV-phage dataset. The performance outcomes of this approach are summarized in Table S5.

	**Recall**	**Precision**	**F1-score**
ESM-2	0.996211	0.985895	0.991027
Original	0.970726	0.994815	0.982623

Table S5. Performance of ESM-enhanced model and PC-based model on test set

The results demonstrate that the ESM-enhanced Transformer marginally outperforms its PC-based counterpart. This improvement likely stems from the richer contextual information captured by ESM embeddings compared to categorical PC labels. However, the ESM-driven approach necessitates GPU acceleration-a resource unavailable to many users-and required approximately 9 hours to generate protein embeddings for the test set. In contrast, Diamond-based PC ID assignment completed the same task in under 10 minutes. These findings highlight the practical trade-off: while the PC-based method sacrifices minimal performance, it demands significantly fewer computational resources and dramatically reduces feature extraction time.

7. COMPARATIVE ANALYSES OF PREDICTED SEQUENCES FROM YANGTZE RIVER DATASET

To calculate BLASTN similarity, we performed BLASTN analyses using a custom reference database compiled from all publicly available RefSeq NCLDV sequences. Each of the 298 MAGs was queried against this database, retaining only the highest-scoring matches (best hits) for downstream analysis. For these alignments, we documented three key parameters: percent identity (sequence similarity), alignment length (number of matching bases), and query coverage (proportion of the MAG sequence aligned to the reference). The results in the supplementary Figure S2 shows that MAGs discovered by GiantHunter exhibit substantial genomic divergence from reference sequences in RefSeq. While a majority of aligned regions display high sequence identity, these matches correspond to short, localized segments, as reflected in the consistently low query coverage values.

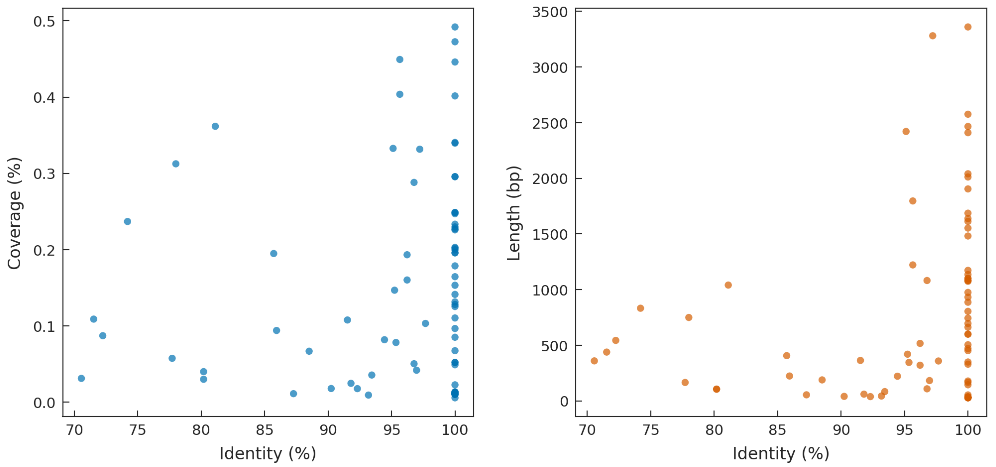

Fig. S2. Alignment metrics of top BLASTN matches. Left: Correlation between percent identity (y-axis) and query coverage (x-axis) across all high-confidence alignments. Right: Relationship between percent identity (y-axis) and alignment length in base pairs (x-axis).

To build a phylogenetic tree, we first collected all the polB genes of NCLDVs in the RefSeq database and use them as reference to recruit polB in the newly identified MAGs. Finally, 66.1% genomes in the RefSeq and 33.5% MAGs were used to construct the phylogenetic tree. Although the marker gene approach will reduce sensitivity, Figure S3 in the supplementary reveals that MAGs identified in our study significantly expand the diversity of the NCLDVs tree. The blue-highlighted clades represent novel lineages of NCLDVs uncovered in this study, underscoring the utility of GiantHunter in discovering novel NCLDVs.

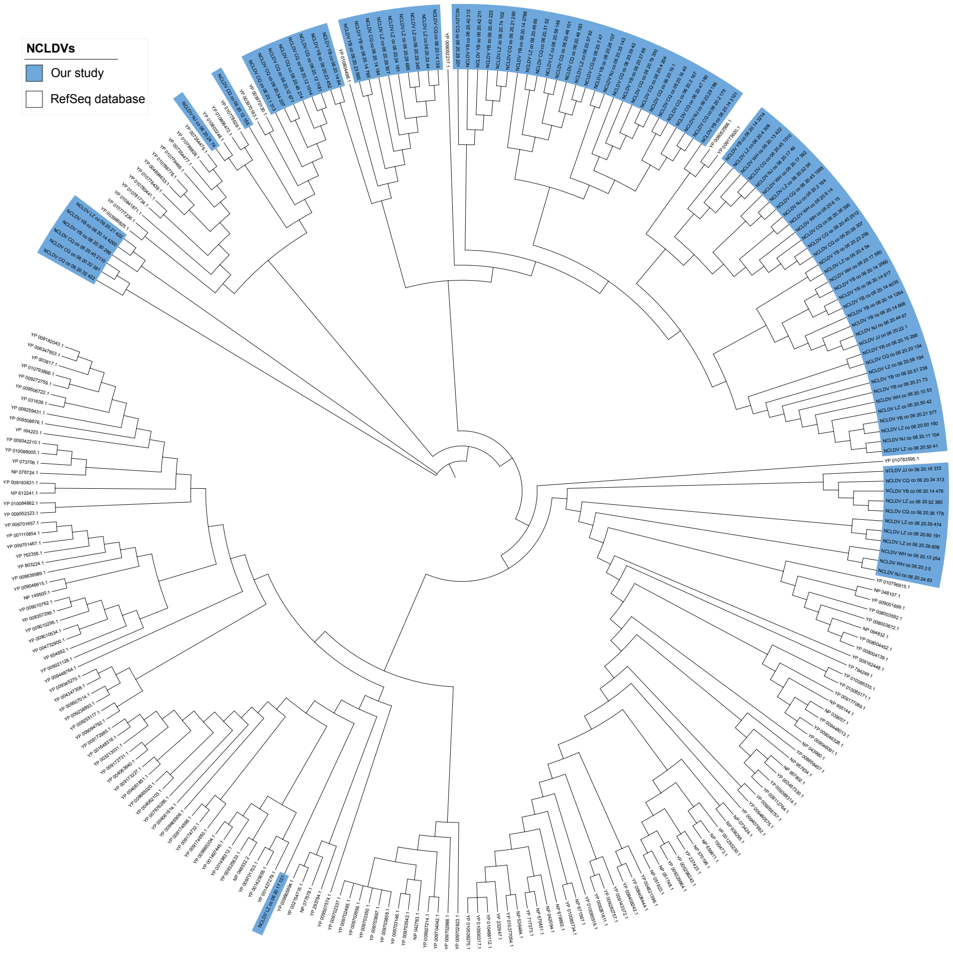

Fig. S3. The phylogenetic tree generated based on the polB marker gene. The blue-highlighted clades represent novel lineages of NCLDVs uncovered in this study, while other clades represent sequences in RefSeq dataset.

REFERENCES

1. A. Vaswani, "Attention is all you need," Adv. Neural Inf. Process. Syst. (2017).
2. Z. Lin, H. Akin, R. Rao, *et al.*, "Evolutionary-scale prediction of atomic-level protein structure with a language model," Science **379**, 1123–1130 (2023).
